# Choledochoduodenostomy rescue with cholangioscopic retrieval of a maldeployed fully covered self-expandable metal stent

**DOI:** 10.1055/a-2787-2305

**Published:** 2026-02-17

**Authors:** Simone Freund, Ulrike W. Denzer

**Affiliations:** 1 61061 Department of Gastroenterology, Endocrinology, Metabolism and Clinical Infectiology, Division of Interdisciplinary Endoscopy, University Hospital of Giessen and Marburg, Campus Marburg, Marburg, Germany

A 75-year-old woman presented at an external hospital for the evaluation of painless jaundice and significant weight loss. Initial imaging raised suspicion of a pancreatic head carcinoma with duodenal infiltration. Endoscopic retrograde cholangiopancreatography (ERCP) performed externally was unsuccessful due to duodenal stenosis, likely secondary to tumor obstruction. An endoscopic ultrasound (EUS)-guided transduodenal puncture of the distal common bile duct (CBD) was performed with the placement of a fully covered self-expandable metal stent (FCSEMS). However, the stent was inadvertently deployed entirely within the CBD without creating a fistulous tract – resulting in intraductal maldeployment with persistent cholestasis. The duodenal puncture site was closed with two hemoclips.

**Fig. 1:**
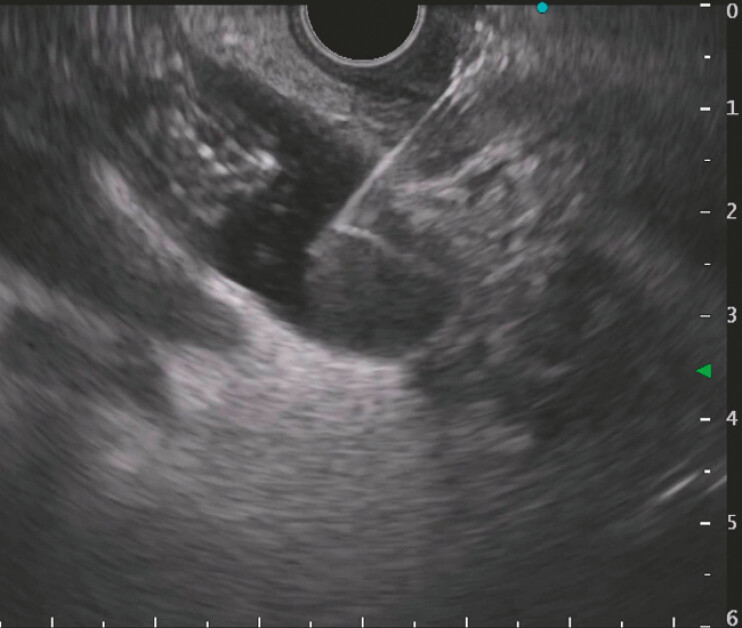
EUS-guided puncture of the distal CBD using a 19G needle. Maldeployed FCSEMS visualized intraductally. CBD, common bile duct; EUS, endoscopic ultrasound; FCSEMS, fully covered self-expandable metal stent.

**Fig. 2:**
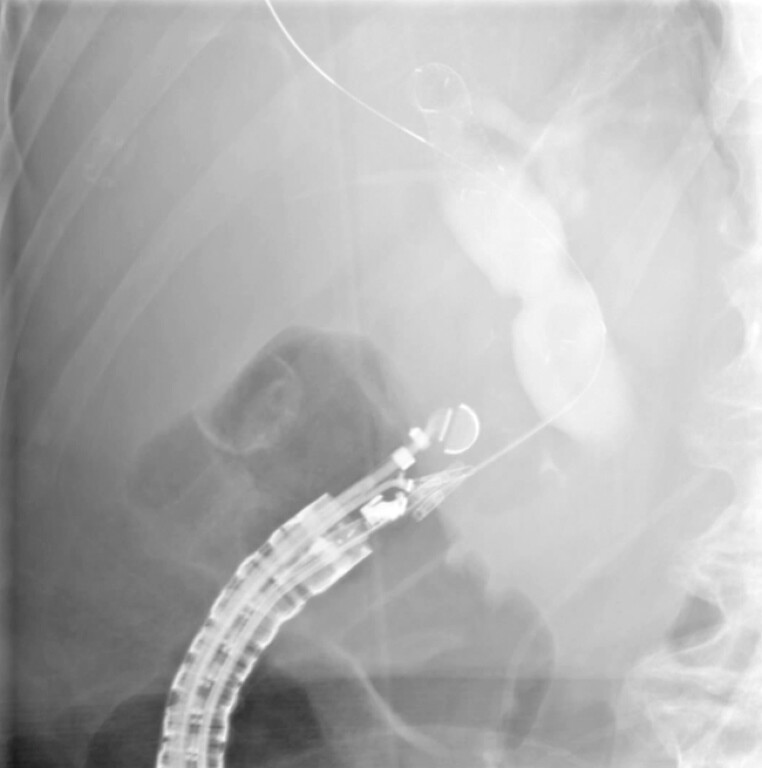
A fluoroscopic view of contrast injection and guidewire insertion into a dilated CBD. CBD, common bile duct.

**Fig. 3:**
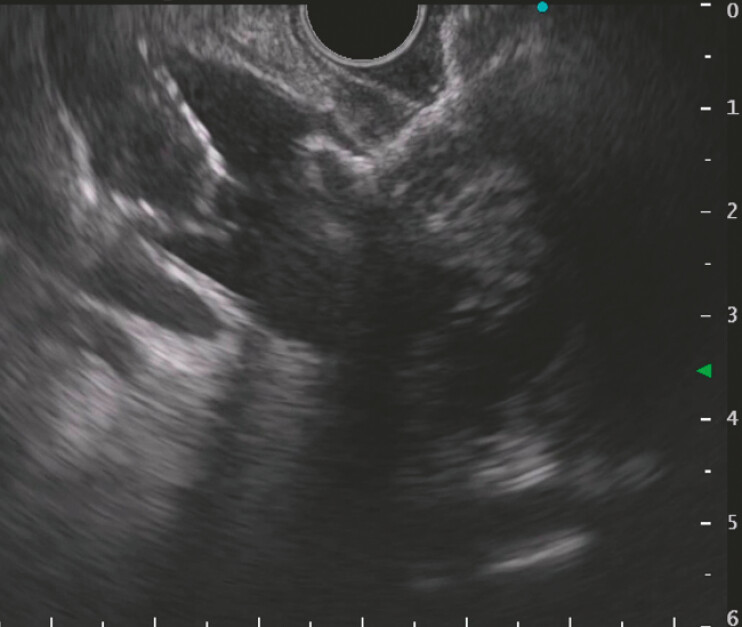
EUS-guided deployment of a 6 × 8 mm Hot LAMS for choledochoduodenostomy. EUS, endoscopic ultrasound; LAMS, lumen-apposing metal stent.

The patient was then referred to our center. Access to the papilla was again not possible due to tumor-related duodenal obstruction. We proceeded with an EUS-guided choledochoduodenostomy (CDS; Video 1 ). The distal CBD was punctured transduodenally using a 19G needle ( Fig. 1 ). After contrast injection, a 0.025-inch guidewire was inserted and advanced intrahepatically under fluoroscopic guidance, bypassing the maldeployed FCSEMS ( Fig. 2 ). A 6 × 8 mm electrocautery-enhanced lumen-apposing metal stent was successfully deployed, resulting in immediate biliary drainage ( Fig. 3 ).

Stepwise endoscopic management of a maldeployed FCSEMS using EUS-guided choledochoduodenostomy and cholangioscopic retrieval through a lumen-apposing metal stent (LAMS) in a patient with malignant biliary obstruction. EUS, endoscopic ultrasound; FCSEMS, fully covered self-expandable metal stent.Video 1

**Fig. 4:**
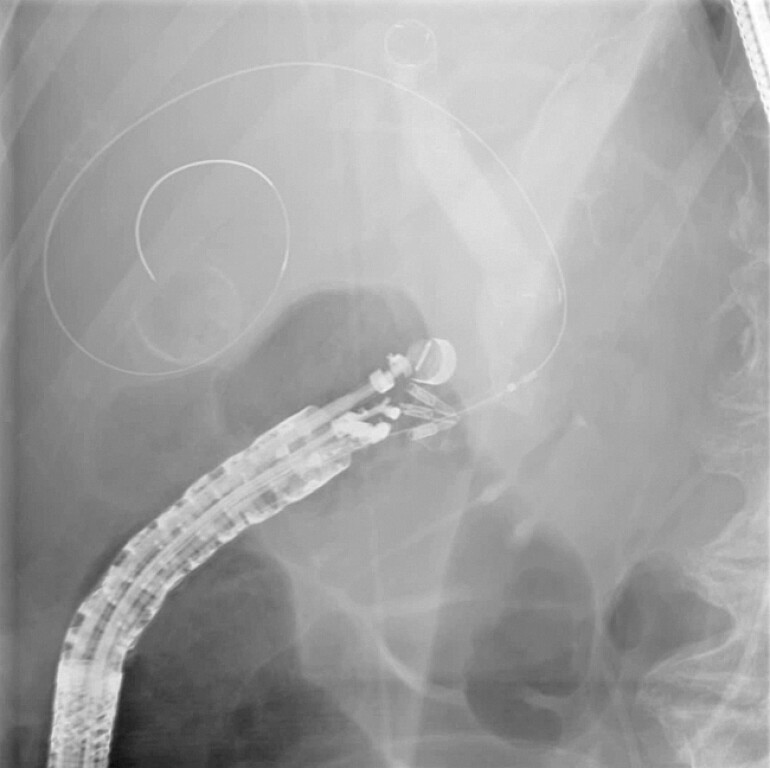
A fluoroscopic view of a fully deployed Hot LAMS during CDS. CDS, choledochoduodenostomy; LAMS, lumen-apposing metal stent.

**Fig. 5:**
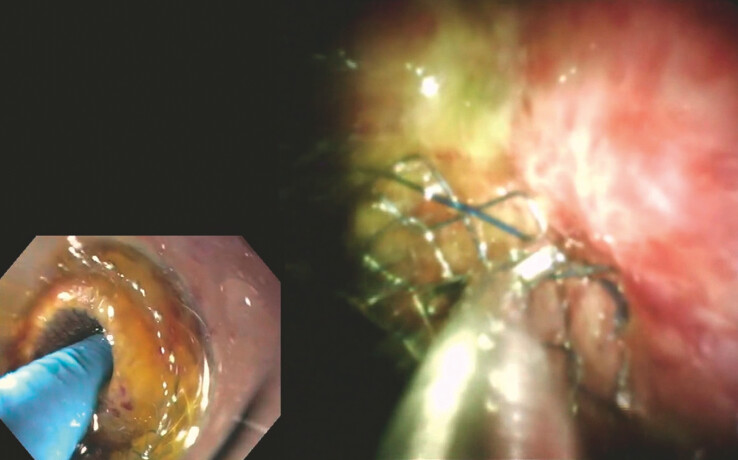
An endoscopic view of the cholangioscopic retrieval of the maldeployed FCSEMS using a forceps through the previously placed LAMS. FCSEMS, fully covered self-expandable metal stent; LAMS, lumen-apposing metal stent.

Five days later, a scheduled reintervention was performed. A therapeutic gastroscope with a transparent hood was advanced to the lumen-apposing metal stent (LAMS), and a 10 Fr cholangioscope was introduced through the LAMS into the CBD. Under direct visualization, the previously maldeployed FCSEMS was identified. Using cholangioscopic biopsy forceps, the stent was grasped at its distal flange and successfully retrieved under direct visualization through the LAMS and the working channel of the endoscope ( Fig. 4 and Fig. 5 ).

This case demonstrates the utility of EUS-guided CDS not only for decompression, but also as a secure access route for advanced intraductal interventions such as cholangioscopic stent rescue – especially in cases where conventional ERCP is not feasible.

Endoscopy_UCTN_Code_CPL_1AK_2AD

